# Identification of Novel Genomic Variations in Susceptibility to Nonsyndromic Cleft Lip and Palate Patients

**DOI:** 10.3390/pediatric13040077

**Published:** 2021-12-08

**Authors:** Kapil Kumar Avasthi, Srinivasan Muthuswamy, Ambreen Asim, Amit Agarwal, Sarita Agarwal

**Affiliations:** 1 Department of Medical Genetics, Sanjay Gandhi Postgraduate Institute of Medical Sciences (SGPGIMS), Lucknow 226014, India; kapilavasthi6@gmail.com (K.K.A.); ambreenasimsiddiqui@gmail.com (A.A.); 2 Department of Life Sciences, National Institute of Technology, Rourkela 769001, Odisha, India; srinimbt@gmail.com; 3 Department of Burn and Plastic Surgery, Vivekananda Polyclinic and Institute of Medical Sciences, Lucknow 226007, India; Dr_amitagarwal78@yahoo.co.in

**Keywords:** NSCL/P, nonsyndromic cleft lip and/or palate, CLP, PMRA, genome-wide association, SNPs

## Abstract

Background: Nonsyndromic cleft lip with or without palate (NSCL/P) is a multifactorial and common birth malformation caused by genetic and environmental factors, as well as by teratogens. Genome-wide association studies found genetic variations with modulatory effects of NSCL/P formation in Chinese and Iranian populations. We aimed to identify the susceptibility of single-nucleotide polymorphisms (SNPs) to nonsyndromic cleft lip with or without palate in the Indian population. Material and Methods: The present study was conducted on NSCL/P cases and controls. Genomic DNA was extracted from peripheral blood and Axiom- Precision Medicine Research Array (PMRA) was performed. The Axiom-PMRA covers 902,527 markers and several thousand novel risk variants. Quality control-passed samples were included for candidate genetic variation identification, gene functional enrichment, and pathway and network analysis. Results: The genome-wide association study identified fourteen novel candidate gene SNPs that showed the most significant association with the risk of NSCL/P, and eight were predicted to have regulatory sequences. Conclusion: The GWAS study showed novel candidate genetic variations in NSCL/P formations. These findings contribute to the understanding of genetic predisposition to nonsyndromic cleft lip with or without palate.

## 1. Introduction

Orofacial clefts (OFCs) are the second most common congenital birth defect in humans [1]. Despite the progress in surgical treatment, the disease has lifelong consequences for the health and social integration of affected people. Worldwide incidence of cleft lip and palate is 1 per 700 live births. However, race, ethnicity, geographical regions, environmental factors and socioeconomic status affect the occurrence of CL/P [2]. The rate of incidences is higher in Asians (European ancestry), and lower in African populations [3]. Proper management of OFCs requires multidisciplinary care and places a significant burden on not only the affected, but also their family, social, and healthcare systems [4]. Therefore, there is great interest in identifying noncleft phenotypic markers for predicting risk in general populations and developing prevention strategies.

OFCs subtypes are defined according to the affected anatomical structures, and can occur as a part of the Mendelian syndrome and isolated nonsyndromic form. Nonsyndromic Orofacial Cleft (NSOFC) is the most common form of Orofacial Clefts, with approximately 70% of cases [1].

Nonsyndromic cleft lip and palate is further classified based on structural morphology—nonsyndromic cleft lip and palate (NSCLP), nonsyndromic cleft lip only (NSCLO) and cleft palate (NSCP) [5]. The causes of NSCLP are multifactorial, the involvement of genetic risk factors, exposure to teratogens and possible genetic–environmental interactions are linked with disease susceptibility. The estimated contribution of all integrated genetic factors to NSCL/P is 90% [6]. M. T. Colobourne et al. stated that CLP and isolated CP cases are governed by environmental factors, and hence it has beocme a necessity explore the interconnection of candidate genes and environmental factors associated with the disease progression. M.T Colobourne et al., in a review article, also highlighted the association of inheritance pattern and genetic loci with this multifactorial disorder [7]. A recent study conducted by Paradowska-Stolarz et al., in 2015, also highlighted that the genetic alternations in the sequences of muscle segment homeobox gene (MSX1) is influenced by environmental factors, thereby leading to the development of deformities including cleft lip palate [8]. Previous studies have also highlighted that interaction of paired box gene 9 (PAX9) with numerous genes playing important roles in different pathways gives rise to such deformities, especially during palate elevation and fusion events [9].

In the past, linkage and candidate gene association studies were performed with traditional genomic techniques. These studies have only identified two common genetic variants that are risk factors for NSCLP. Risk loci identified in the meta-analysis of linkage studies are interferon regulatory factor 6 IRF6 (1q32.2) and forkhead box E1 FOXE1 (9q22.33) [10,11]. Several significant genomic findings in NSCLP have arisen from high-throughput tools such as genome-wide association (GWAS) in the recent genomic era. Studies so far have identified a total of 40 genome-wide significant risk loci for NSLC/P in various populations. Of these, 37 loci were detected by GWAS, metadata analysis of GWAS, or subsequent studies [12,13,14,15,16,17,18,19,20,21,22,23,24]. In the genomic era, new sequencing techniques including whole-exome sequencing have identified cadherin-1 (CHD1) and some other new susceptible genes in NSCLP [25,26,27].

The racial differences must be considered for the fundamental identification of genomic susceptibility to complex diseases in a multiethnic population [28]. There is no GWAS study that has been performed and reported on the Indian population. An advancement in genomic array techniques, the Axiom-Precision Medicine Research Array (PMRA) is comprehensive genotyping array based on South Asian and East Asian populations, containing more than 750,000 markers, including common and rare disease-associated variants catalogued in public databases such as the GWAS catalog and ClinVar (Thermo Fisher Scientific, Walthman, MA, USA). In the present study, we tried to identify the genetic variants predicting nonsyndromic cleft lip and palate (NSCL/P) in the Indian population. The best of our knowledge, this is the first GWAS report that aims to provide a better understanding of NSCLP formation with Asia-specific PMRA.

## 2. Material & Methods

### 2.1. Sample Collection

In this study, clinically confirmed nonsyndromic cleft lip and palate (N = 86; age of patients ranging from 0 to 2 years) patients and age–sex-matched patients ranging from 0 to 2 years, including male and female healthy controls (N = 10) were recruited for the study, after signing an informed consent form. Patients were recruited from Medical Genetics OPD, Sanjay Gandhi Postgraduate Institute of Medical Sciences, Lucknow (India) and Plastic, craniofacial & microsurgery OPD, Vivekananda polyclinic & Institute of Medical Sciences, Lucknow (UP). The exclusion criteria for NSCLP cases were patients with any other history of developmental disorder, syndromic forms of NSCLP (e.g., eye, brain, limb anomalies and cardiac defects). The healthy controls were Indian-origin children without family history of orofacial cleft.

The study was approved by the Institutional Medical Research Ethics Committee of the Sanjay Gandhi Postgraduate Institute of Medical Sciences, India, (2018-107-EMP-EXP), and a written informed consent was taken from the guardians.

Genotyping was conducted for 86 cases and 10 control samples using Affymetrix PMRA array.

### 2.2. DNA Isolation

Genomic DNA was extracted from 3 mL peripheral venous blood samples of patient and controls, with DNA extracted by using standard phenol–chloroform method (Nasiri, 2005). The quality of DNA was assessed on 1% agarose gel electrophoresis, and quantity of DNA was measured by NanoDrop^TM^.

### 2.3. Axiom^TM^ PMRA Assay

The NSCLP patient samples were genotyped using the Axiom^TM^ Asia-precision medicine Research Array (PMRA) kit (Thermo Fisher Scientific, Waltham, MA, USA) for genotyping, using the Affymatrix GeneTitan platform. It contains over 750,000 SNPs including 50,000 novel markers covering South and East Asian populations based on the human genome version 19 (build 37). Target probes were prepared using high-quality DNA (20 ng) in each well according to the standard protocol of the Affymatrix Axiom 2.0 assay. In addition, DNA samples were amplified, fragmented and hybridised on a chip, followed by single-base expansion via DNA ligation and signal modification. Samples were stained and scanned using Affymatrix GeneTitan multichannel instrument.

### 2.4. Quality Control

Axiom Analysis suite version 1.0 (http://media.affymetrix.com/support/downloads/manuals/Axiom_analysis_suite_user_guide.pdf, Last accessed 13 September 2020) was used for detection of allele-specific SNPs, and a best-practice workflow was used for quality-control analysis of the dataset. A total of 845,858 out of 888,799 variants, including small insertions/deletions (indels), passed the quality-control test with a genotype call rate of ≥ 95%. Samples with DQC < 0.82 and QC call rate < 97% were not considered for further analysis. SNP QC was performed under default parameters for humans, that is, cr-cutoff ≥ 95; fld-cutoff ≥ 3.6; het-so-cutoff ≥ 0.1; het-so-otv-cutoff ≥ 0.3; hom-ro-1-cutoff ≥ 0.6; hom-ro-2-cutoff ≥ 0.3.

### 2.5. Allele Calling and Data Analysis

The genotyping workflow was used to perform genotyping of the imported .CELL files. Finally, the summary-only workflow was used to summarise the intensity details of the probe set for use in further analysis tools. Plink software was used to perform the GWAS analysis and Haploview was used for generating Manhattan plot.

## 3. Results

A total of eight hundred ten thousand nine hundred six single-nucleotide variants were genotyped, with a genotyping rate of 0.99. The data were filtered for missing genotypes, and one lakh two thousand eight hundred eighty-seven SNPs were removed. Similarly, three lakhs seventy-one thousand eight hundred and ten SNPs with a minor allele frequency of less than 0.01 and 278 SNPs deviating Hardy–Weinberg equilibrium, with a *p*-value of 0.001, were removed from further analysis. Three lakhs thirty-five thousand nine hundred seventy-one SNPs passed the filtering for analysis.

**Figure 1:**
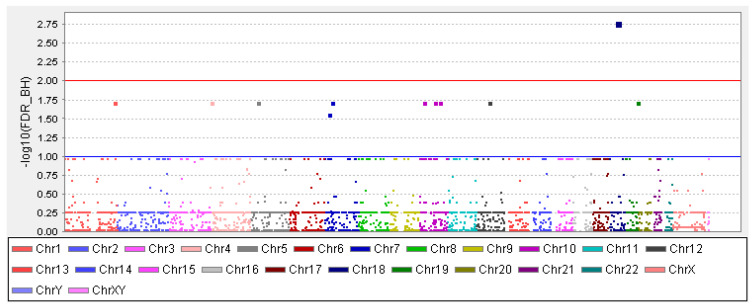
Manhattan plot with a blue line and red line indicating the threshold for genome-wide significance. The *x*-axis has the chromosome numbers and -log 10 FDR–BH (false discovery rate–Benjamini and Hochberg) adjusted *p*-value in the *y*-axis.

GWAS results: Of three lakhs thirty-five thousand nine hundred seventy-one SNPs analysed for their genetic association with the OFCs, rs36019844 showed a significant association (FDR Benjamini and Hochberg *p*-value of 0.001688, Figure 1). Additionally, 13 other SNPs were also found to be associated with the OFC (Table 1). Among these 13 SNPs, 6 of them were in the intronic region of the genes ZNF503 (rs74146603), SLC35F3 (rs146076295), ENTPD1 (rs117864318), PIP4K2A (rs80087712), and EHBP1 (rs115646634).

Since significant SNPs are found in the intronic and intergenic region, we predicted a gene regulatory element’s presence using RegulomeDB. Out of 14 SNPs, 8 were predicted to have regulatory sequences in them, as given in Table 2. The list of deciphered morbid genes that are located in the 1 mb region before and after the significant SNPs are shown in Table 3.

## 4. Discussion

Orofacial clefts are a developmental anomaly that occur during pregnancy. The body tissue that makes up the lips and palate fails to join completely before birth. Such failure results in a small-to-large opening originating in the lip and going through the nose. The clefting can be unilateral or bilateral. The clefting in newborns disturbs their feeding and speaking ability.

Though numerous studies are available to explain the aetiology of the CLP, we do not have a clear understanding of it. This poor understanding is because of the multifactorial nature of the condition. To a large extent, a genetic cause for the syndromic form of CLP is identifiable. However, identifying the causative factor in isolated cases is challenging even though numerous genetic variants have been attributed to CL/P [29]. Therefore, this study was undertaken to find the genetic variants underlying the isolated cases of CL/P using Affymetrix PMRA array.

In this study, we performed a genome-wide analysis of SNPs using the Affymetrix PMRA array in a cohort of 86 cases and 10 control subjects. We identified 14 SNPs with statistical significance that are distributed across chromosomes 1, 2, 4, 5 (2 SNPs), 7 (2 SNPs), 10 (3 SNPs), 12, 17, 18 and 19. Except for rs80357922, all other variants were located in the intronic or intergenic region.

The most statistically significant signal was obtained for rs36019844, a variant located in MIR924HG, or Long Intergenic Non-Protein-Coding RNA gene. Unfortunately, until now, no regulatory function of MIR924HG has been characterized. We tried to identify any gene that was located 1 MB upstream and downstream of the identified SNP. The MOCOS gene was found, and it encodes for Molybdenum Cofactor Sulphur-transferase enzyme, involved in the activation of the xanthine dehydrogenase (XDH) and aldehyde oxidase (AO) enzymes. Xanthinuria Type II and Xanthinuria are diseases associated with the MOCOS gene.

Interestingly, the BRCA1 gene showed a significant association, emphasizing that subjects born with CLP are susceptible to cancer and have shorter lifespan [30,31,32]. The genes that regulate development are frequently associated with cancers and variations in genes critical functions, such as DNA damage and repair, including BRCA1, increase susceptibility to NSCLP [33,34]. In a Denmark-based study, it was reported that breast and brain cancers are frequent among female and primary lung cancer among male CLP subjects [35]. A recent study by Li A. et al., in 2019, attempted to investigate the association between transcription factors, miRNA’s and cleft lip genes by constructing miRNA–TF coregulatory networks, and identified 10 crucial genetic markers in the signalling pathways regulating pluripotency of stems cells. The study further concluded by highlighting the critical role of miRNAs, namely hsa-mir-27b and hsa-mir-497, in triggering the expression of the Wnt pathway, which is involved in the occurrence of cleft lip [36].

Most identified SNPs are in the intronic and intergenic region, and we tried to find out whether such regions have regulatory elements that may act as cis-regulatory elements for nearby genes. RegulomeDB score was estimated for each of the SNPs. Four SNPs (rs113361480, rs10254958, rs74146603 and rs80087712) had a rank of 3a, which signifies that they are probably a TF-binding site, DNase peak and a motif element, while rs146076295, rs116146139, rs80357922 and rs8102243 had a rank of 4, which indicates that these SNPs are associated with transcription factor binding and DNase peaks.

## 5. Conclusions

Our study demonstrated that rs36019844 at loci 18q12.2 showed the highest statistical significance for its association with the orofacial cleft. Following which, 13 other SNPs (Table 1) showed significance. However, our study also highlighted the association of BRACA1 gene with CLP subjects, thus further providing the emphasis on the risk of cancer development in such cases.

### Limitation of Study

The current study is region-centred, however, to establish strong evidence for the association, further detailed analysis of a larger cohort in different ethnic groups is essential.

## Figures and Tables

**Table 1:** List of SNPs that showed a significant association with an orofacial cleft in GWAS.

S. No.	CHR	SNP	UNADJ	Gene	Loci	Region	FDR_BH
1.	18	rs36019844	2.08E-09	MIR924HG	18q12.2	Intron	0.001688
2.	10	rs74146603	1.68E-07	ZNF503	10q22.2	Intron	0.01943
3.	4	rs7680206	1.68E-07	Noncoding	4p16.1	Intron	0.01943
4.	7	rs10254958	2.46E-07	Noncoding	7p14.2	Intron	0.01943
5.	12	rs113361480	2.46E-07	Noncoding	12q14.1	Intron	0.01943
6.	1	rs146076295	2.46E-07	SLC35F3	1q42.2	Intron	0.01943
7.	19	rs8102243	2.46E-07	Noncoding	19q13.42	Intron	0.01943
8.	10	rs117864318	2.46E-07	ENTPD1	10q24.1	Intron	0.01943
9.	5	rs116146139	2.46E-07	SUB1	5p13.3	Intron	0.01943
10.	10	rs80087712	2.46E-07	PIP4K2A	10p12.2	Intron	0.01943
11.	2	rs115646634	2.88E-07	EHBP1	2p15	Intron	0.01943
12.	5	rs144839912	2.88E-07	Noncoding	5q33.2	Intron	0.01943
13.	17	rs80357922	4.59E-07	BRCA1	17q21.31	NM_007299.4: c.787+1209del	0.02741
14.	7	rs12666118	4.73E-07	Noncoding	7p21.1	Intron	0.02741

Abbreviations: CHR—chromosome; SNP—single-nucleotide polymorphism; UNADJ—p- value; FDR_BH—false discovery rate Benjamini & Hochberg; MR924HG—micro-RNA 924 host gene; ZNF503—zinc finger protein 503; SLC35F3—solute carrier family 35 member F3; ENTPD1—Ectonucleoside Triphosphate Diphosphohydrolase 1; PIP4K2A—phosphatidylinositol-5-phosphate 4-kinase type 2 alpha; EHBP1—EH Domain Binding Protein 1; BRCA1—Breast cancer type 1.

**Table 2:** RegulomeDB score for the SNPs that showed significance in the GWAS analysis.

S. No.	Chromosome Coordinates	dbSNP IDs	Rank	Score
1.	Chr12:61874709-61874710	rs113361480	3a	0.5497
2.	Chr7: 35575753-35575754	rs10254958	3a	0.57268
3.	Chr10:77123420-77123421	rs74146603	3a	0.70695
4.	Chr10:22832058-22832059	rs80087712	4	0.97433
5.	Chr1:234297126-234297127	rs146076295	4	0.60906
6.	Chr5:32535226-32535227	rs116146139	4	0.60906
7.	Chr17:41245550-41245551	rs80357922	4	0.60906
8.	Chr19:56039472-56039473	rs8102243	4	0.60906
9.	Chr7:19870964-19870965	rs12666118	5	0.0
10.	Chr5:153209523-153209524	rs144839912	5	0.008
11.	Chr4: 6319865-6319866	rs7680206	5	0.13454
12.	Chr10:97485829-97485830	rs117864318	6	–
13.	Chr18:37254233-37254234	rs36019844	6	-
14.	Chr2:63019086-63019087	Rs115646634	7	-

Abbreviations: CHR—chromosome; SNP—single-nucleotide polymorphism; dbSNP—databank single-nucleotide polymorphism.

**Table 3:** List of deciphered morbid genes that are located in the 1 mb region before and after the significant SNPs.

S.No.	Chromosome Coordinates	SNPs	Morbid Genes in +-1mb region of SNPs
1.	chr12:61874709-61874710	rs113361480	USP15, PPM1H
2.	chr7:35575753-35575754	rs10254958	TBX20, NSPR1, ANLN
3.	chr10:77123420-77123421	rs74146603	KCNMA1, POLR3, RPS24, LRMDA
4.	chr10:22832058-22832059	rs80087712	PTF1A
5.	chr1:234297126-234297127	rs146076295	COA6, IRF2BP2
6.	chr5:32535226-32535227	rs116146139	TARS1, NPR3
7.	chr17:41245550-41245551	rs80357922	Too many
8.	chr19:56039472-56039473	rs8102243	TNNT1, TNNI3, DNAAF3
9.	chr7:19870964-19870965	rs12666118	TWIST1
10.	chr5:153209523-153209524	rs144839912	None
11.	chr4:6319865-6319866	rs7680206	EVC2, EVC, WFS1
12.	chr10:97485829-97485830	rs117864318	HOGA1, ZFYVE27, HPS1
13.	chr18:37254233-37254234	rs36019844	MOCOS
14.	chr2:63019086-63019087	rs115646634	EHBP1, WDPCP, MDH1, UGP2

Abbreviations: CHR—chromosome; SNP—single-nucleotide polymorphism; USP15—ubiquitin specific peptidase 15; PPM1H—Protein Phosphatase, Mg2+/Mn2+ Dependent 1H; TBX20—T-Box Transcription Factor 20; NSPR1—neuropeptide S receptor 1; ANLN—anillin actin binding protein; KCNMA1—potassium calcium-activated channel subfamily M alpha 1; POLR3—RNA polymerase III-related leukodystrophy; RPS24—ribosomal protein S24; LRMDA—leucine-rich melanocyte differentiation associated; PTF1A—pancreas-specific transcription factor 1a; COA6—Cytochrome c oxidase assembly factor 6; IRF2BP2—interferon regulatory factor 2 binding protein 2; TARS1—Threonyl-TRNA Synthetase 1; NPR3—natriuretic peptide receptor 3; TNNT1—Troponin T1, Slow Skeletal Type; TNNI3—troponin I3, cardiac type; DNAAF3—dynein axonemal assembly factor 3; TWIST1—Twist Family BHLH Transcription Factor 1; EVC2 —EvC Ciliary Complex Subunit 2; WFS1—wolframin; HOGA1—4-hydroxy-2-oxoglutarate aldolase; ZFYVE27—zinc finger FYVE-type containing 27; HPS1—Hermansky–Pudlak syndrome 1 protein; MOCOS—molybdenum cofactor sulfurase; EHBP1—EH Domain Binding Protein 1; WDPCP—WD Repeat-Containing Planar Cell Polarity Effector; MDH1—malate dehydrogenase 1; UGP2—UDP-glucose pyrophosphorylase 2.

## Data Availability

Data are available upon reasonable request.
